# Persistence of Transmitted HIV-1 Drug Resistance Mutations Associated with Fitness Costs and Viral Genetic Backgrounds

**DOI:** 10.1371/journal.ppat.1004722

**Published:** 2015-03-23

**Authors:** Wan-Lin Yang, Roger D. Kouyos, Jürg Böni, Sabine Yerly, Thomas Klimkait, Vincent Aubert, Alexandra U. Scherrer, Mohaned Shilaih, Trevor Hinkley, Christos Petropoulos, Sebastian Bonhoeffer, Huldrych F. Günthard, the Swiss HIV Cohort Study (SHCS)

**Affiliations:** 1 Division of Infectious Diseases and Hospital Epidemiology, University Hospital Zurich, University of Zurich, Zurich, Switzerland; 2 Institute of Medical Virology, University of Zurich, Zurich, Switzerland; 3 Laboratory of Virology and AIDS Center, Geneva University Hospital, Geneva, Switzerland; 4 Department Biomedicine—Petersplatz, University of Basel, Basel, Switzerland; 5 Division of Immunology and Allergy, Centre Hospitalier Universitaire Vaudois and University of Lausanne, Lausanne, Switzerland; 6 Institute of Integrative Biology, ETH Zurich, Zurich, Switzerland; 7 Monogram Biosciences, South San Francisco, California, United States of America; University of North Carolina at Chapel Hill, UNITED STATES

## Abstract

Transmission of drug-resistant pathogens presents an almost-universal challenge for fighting infectious diseases. Transmitted drug resistance mutations (TDRM) can persist in the absence of drugs for considerable time. It is generally believed that differential TDRM-persistence is caused, at least partially, by variations in TDRM-fitness-costs. However, *in vivo* epidemiological evidence for the impact of fitness costs on TDRM-persistence is rare.

Here, we studied the persistence of TDRM in HIV-1 using longitudinally-sampled nucleotide sequences from the Swiss-HIV-Cohort-Study (SHCS). All treatment-naïve individuals with TDRM at baseline were included. Persistence of TDRM was quantified via reversion rates (RR) determined with interval-censored survival models. Fitness costs of TDRM were estimated in the genetic background in which they occurred using a previously published and validated machine-learning algorithm (based on *in vitro* replicative capacities) and were included in the survival models as explanatory variables.

In 857 sequential samples from 168 treatment-naïve patients, 17 TDRM were analyzed. RR varied substantially and ranged from 174.0/100-person-years;CI=[51.4, 588.8] (for 184V) to 2.7/100-person-years;[0.7, 10.9] (for 215D). RR increased significantly with fitness cost (increase by 1.6[1.3,2.0] per standard deviation of fitness costs). When subdividing fitness costs into the average fitness cost of a given mutation and the deviation from the average fitness cost of a mutation in a given genetic background, we found that both components were significantly associated with reversion-rates.

Our results show that the substantial variations of TDRM persistence in the absence of drugs are associated with fitness-cost differences both among mutations and among different genetic backgrounds for the same mutation.

## Author Summary

The evolution of resistance is a universal challenge in antimicrobial chemotherapy. A key driver of resistance is that drug resistance mutations often persist even in the absence of drugs and despite the fact that resistance mutations are often associated with reduced pathogen replication (“fitness costs”). Such persistence may occur because fitness costs are low, especially if they are compensated by additional mutations in their “genetic background”. Here we assessed the role of fitness-cost and the genetic background for resistance in a real-world epidemiological setting by studying the persistence behavior of transmitted antiretroviral resistance mutations of HIV. This persistence behavior was associated with the predicted fitness cost of a given resistance mutation in the particular genetic background in which it occurred. We found that persistence behavior varied strongly across both mutation types and genetic backgrounds and that persistence was significantly associated with predicted fitness costs. In particular we found that even mutations of the same type tended to persist longer if they occurred in a genetic background where they caused weak fitness costs. Overall our results underline the variability of persistence behavior as well as the important role of fitness costs and the genetic background in the evolution of antimicrobial resistance.

## Introduction

Drug-resistant pathogens represent one of the major public health and clinical challenges in infectious diseases (http://www.who.int/drugresistance/en/). It is an almost universal observation that as soon as a chemotherapeutic agent against a given pathogen is introduced, resistant pathogen strains emerge, which reduce the clinical benefits conferred by that agent. One crucial obstacle in curbing drug resistance is that once it has emerged it often persists even in the absence of drug pressure. The central concept here is pathogen fitness: whereas the resistant pathogen has a very strong advantage over the sensitive one in the presence of drug pressure, its disadvantages in the absence of treatment are typically weaker and can be compensated by other mechanisms such as compensatory mutations or selection at linked loci. Despite this key role of pathogen fitness for a conceptual understanding of the spread and persistence of drug resistance, real-world epidemiological examples documenting its role are rare. An ideal opportunity to assess this role of fitness is provided by the dynamics of antiretroviral resistance in HIV-1.

In the case of HIV, combinations of modern anti-retroviral treatment (ART) have successfully reduced the morbidity and mortality of HIV-1 infected individuals [1]. Though drug resistance prevalence has been shown to decrease or to stabilize in various industrialized countries due to successful ART, it still remains a major concern jeopardizing treatment success [2,3].

Transmission of a drug-resistant virus has been observed in most countries where ART is available [4–10]. After transmission, viruses with transmitted drug resistance mutations (TDRM) persist either as the dominant species or as minority variants, which are difficult to detect by population sequencing techniques [11–17]. Consequently, patients harboring TDRM have a higher chance to fail their first-line therapy [12,18–20].

Several studies have illustrated that the persistence time of individual TDRM in the absence of drug pressure exhibits substantial variance [11,13,15,17,21,22]. Persistence times have been suggested to be associated with fitness costs [18], which are typically measured as the reduction of replicative capacity of the virus caused by a given mutation [21]. It is generally assumed that transmitted drug-resistant viruses revert more rapidly to wild-type viruses if the fitness is reduced to a larger extent by the TDRM (high fitness cost) because then reversion of TDRM confers correspondingly high fitness gains [23]. Several studies have measured the fitness of some specific TDRM using phenotypic replicative capacity assays [6,17,21]. However, evidence for the impact of such fitness costs on the dynamics of TDRM at an *in vivo* and epidemiological level is largely lacking. Here, we aimed to determine the persistence times of TDRM in an epidemiological approach *in vivo* and to determine whether these persistence times depend on the fitness costs of TDRM.

## Methods

### Study population

The SHCS is a prospective, nationwide, clinic-based study including a biobank. The SHCS is very representative of the HIV epidemiology in Switzerland; it includes at least 53% of all HIV cases ever diagnosed in Switzerland, 72% of all patients receiving ART, and 69% of the nationwide registered AIDS cases [24,25]. Since 1996, the SHCS includes approximately 85% of the newly diagnosed HIV infected individuals in Switzerland. This number was obtained when we compared the estimated numbers of newly diagnosed HIV cases published by the Swiss Federal Office of Public Health to the numbers of patients enrolled in the SHCS annually since 1996. Genotypic resistance data stem from routine clinical testing and from systematic retrospective sequencing before routine genotyping was introduced (over 11000 sequences were retrospectively generated). Genotyping is performed by four laboratories in Switzerland authorized by the Federal Office of Public Health. All laboratories perform population-based sequencing of the full protease gene and at least codons 28–225 of the reverse transcriptase gene using commercial assays such as Viroseq Vs.1 PE Biosystems; Virsoseq Vs. 2, Abbott AG; VircoTYPE HIV-1 Assay, Virco Lab or in-house methods [4] and has participated in the yearly quality control evaluation by the Agence Nationale de la Recherche du SIDA(ANRS) since 2002. All sequences are stored the SHCS drug-resistance database using SmartGene’s Integrated Dababase Network System (SmartGene, Zug, Switzerland, IDNS version 3.6.3) [12]. For details on the sequencing procedure, see [12]. To increase coverage, we have systematically selected all treatment-naïve individuals carrying TDRM and retrieved their sequential plasma samples before therapy from the SHCS biobank.

For this study we considered genotypic resistance test (GRT) performed for a patient when being treatment-naïve. All sequential GRTs were included for individuals having ≥ 2 GRTs and harboring TDRM at baseline before ever starting any antiretroviral therapy.

TDRM was defined according to the WHO surveillance list of transmitted HIV drug resistance [11]. We studied mutations to the major three drug classes: nucleoside and nucleotide analogue reverse transcriptase inhibitors (NRTIs), protease inhibitors (PIs), and nonnucleoside reverse transcriptase inhibitors (NNRTIs). Additionally, we excluded 17 potential super-infections based on phylogenetic distance and the lack of phylogenetic clustering. Finally, since TDRM in HIV-1 CTL epitopes can disrupt binding to the HLA allele and such CTL-escape may essentially influence the reversion dynamics, we screened the list of optimal HIV-1 CTL epitopes (according to the Los Alamos HIV database, http://www.hiv.lanl.gov/content/immunology/pdf/2013/optimal_ctl_article.pdf) for epitopes containing TDRM and excluded from our analysis those mutations that disrupted binding to the epitope according to NetMHCcons (http://www.cbs.dtu.dk/services/NetMHCcons/).

### Ethics statement

The SHCS, enrolling HIV-infected adults aged ≥ 16 years old, has been approved by ethics committees of all participating institutions. The data collection was anonymous and written informed consent was obtained from all participants [24].

### Survival analysis

Our goal was to assess systematically the persistence of TDRM in the absence of drug pressure. In particular we considered the persistence across different mutations and viral genetic backgrounds (for a given mutation occurring in a given virus, the viral genetic background is given by the entire amino acid sequence in which this mutation is observed). To allow inter-patient comparisons we included TDRM that were present in at least five individuals at baseline.

We quantified the persistence via calculating reversion rates of individual TDRMs. Reversion of a TDRM was defined as an event at which a TDRM becomes undetectable by population sequencing assays. In other words, a TDRM has reversed when the HIV variant carrying that TDRM has decreased to the level below the detection limit of population sequencing assays (∼20–30% [26]). Therefore, reversion is not necessarily always to wild type. We fitted our data with an interval-censored survival model using exponential waiting times. We chose an interval-censored model because the data did not allow to determine the exact time point of reversion; instead a GRT not detecting a given resistance mutation preceded by a GRT with that mutation informs that the reversion event must have occurred in the time interval between those two tests.

Our results were expressed with 95% CI and two-sided p-values with p<0·05 being statistically significant. We analyzed our data with Stata 13.1 SE (StataCorp, Texas, USA).

### Estimation of fitness costs of TDRM

We estimated fitness costs based on a previously published approach to predict HIV replicative fitness from amino acid sequences [27]. This approach uses a machine-learning algorithm (ridge regression) trained on >70000 data points, each consisting of a *pol*-amino-acid sequence and an *in vitro* replicative capacity. Specifically, the algorithm predicts replicative capacity (pRC) from an amino acid sequence by a quadratic fitness model of the form

$$pRC\left(x\right)=\sum_{ij}M_{ij}x_{i}x_{j}$$

where *x*_*i*_ denotes the presence (1) or absence (0) of a given mutation *i* and *M_ij_* the epistatic effects (i<j) and the main effects (i = j) characterizing the fitness landscape. These coefficients were derived in [27] by fitting the model to the >70000 data points. Since the number of parameters of the above model exceeds the number of data points, this model was fitted using an approach based on ridge regression. In essence, in this approach the data set was split into a “training”, “training-test”, and “true-test” data set. Then assuming a given penalty weight for model parameters, the model parameters are determined such that for the “training” data set, the sum of squared residuals plus the sum of squares of parameters times the penalty weight are minimized. In this specific case the approach was modified to a generalized linear ridge regression to take the non-normal error structure into account. The model was evaluated on the “test-training” data set, and the penalty weight was determined such that the predictive power on the “test-training” test was optimized. This final model was then evaluated on the “true-test” data set (which was used neither in deriving the model parameters nor in determining the penalty weight). Details on the method and validation on *in vitro* and clinical data can be found in [27] and [28]. Using this model, we estimated the fitness cost of a mutation in a given genetic background as follows. If *A* denotes the partial *pol*-amino-acid sequence (first 404 amino acid used in the reference [27]) with a given resistance mutation *m* and *A’* the same amino acid sequence but with the mutation reverted to its wild-type allele, then the fitness cost of the mutation *m* in the background *A* can be estimated as

c(m,A)=pRC(A)−pRC(A′).

A negative fitness cost was set to zero.

The impact of this fitness cost was assessed in univariable and multivariable versions of the interval-censored model. The multivariable models were adjusted for whether a given TDRM was present as a mixture with another amino acid at this position. Specifically, this was considered to be the case if the nucleotide sequence coding for this mutation contained at least one ambiguous nucleotide that affects the amino acid encoded.

## Results

### Study population

From 7920 treatment-naïve patients enrolled in the SHCS from May 1995 to February 2013, we could identify 987 sequential GRTs from 197 patients, who had ≥ 2 GRT while being treatment-naïve and presented with ≥ 1 TDRM at baseline. See S1 Table for all types and numbers of mutations and reversions observed from these 197 patients. The criterion that a given mutation must have been present in at least 5 individuals at baseline reduced the number of sequential GRTs and patients to 857 and 168, respectively.

From our studied population most individuals were male (80%), white (87.5%), and infected with subtype-B viruses (81.5%; Table 1). The median (IQR) number of GRT performed per person was 7 (4, 11) and the median (IQR) of test interval was 193 (170, 243) days. Baseline CD4 count was relatively high (494 [347, 656]), suggesting that patients were tested relatively early on after infection. 60.1% of patients had a single mutation detected at their first GRT. Detailed patient characteristics were shown in Table 1.

**Table 1:** Basic characteristics of study population.

	**No. of patients (%) or Median (IQR)**
**Patients included**	168
**Age at baseline**	35 (30.5, 40)
**Gender**	
**Male**	133 (79.2)
**Female**	35 (20.8)
**Ethnicity**	
**White**	147 (87.5)
**Black**	11 (6.5)
**Others / Unknown**	10 (6.0)
**Transmission route**	
**MSM (Male Homosexual)**	83 (49.4)
**Heterosexual**	47 (28.0)
**Intravenous drug users**	33 (19.6)
**Unknown**	5 (3.0)
**Subtype**	
**B**	137 (81.5)
**Non-B**	26 (15.5)
**Non-classified**	5 (3.0)
**Viral load at baseline (log_10_ copies/ml)** ^**1**^	4.4 (3.6, 4.9)
**CD4 count at baseline (cells/mm^3^)** ^**2**^	494 (347, 656)
**No. of mutations at baseline**	
**1**	101 (60.1)
**2**	34 (20.2)
**≥ 3**	33 (19.6)
**Mutations at baseline resistant to**	
**NRTI**	101 (60.1)
**PI**	51 (30.4)
**NNRTI**	47 (28.0)
**No. of resistant classes** ^**3**^ at baseline	
**1**	142 (84.5)
**2**	21 (12.5)
**3**	5 (3.0)
**Test interval in days**	193 (170, 243)
**Number of GRT performed**	7 (4, 11)

1 within 30 days before / after the first resistance test, N = 151 (90%)

2 within 30 days before /after the first resistance test, N = 157 (93%)

3 having ≥ 1 resistant mutations of a drug class

### Reversion rate of individual TDRM varies

**Fig 1:**
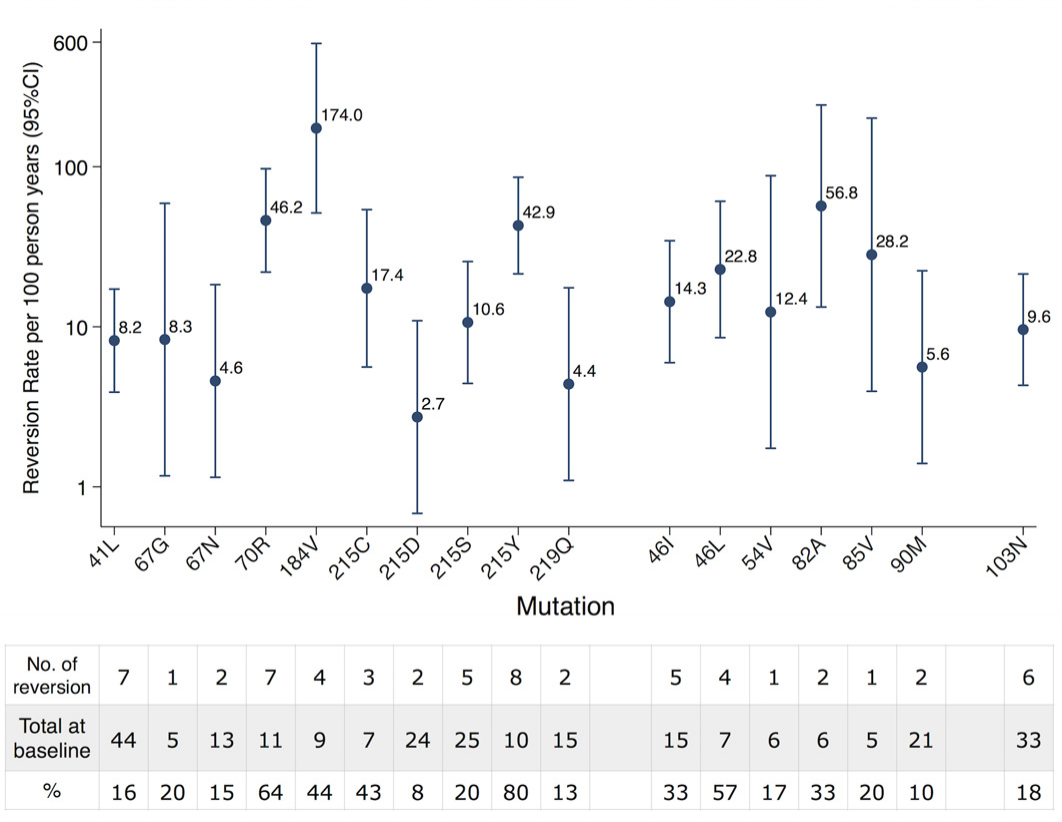
Reversion rate of individual TDRM. Reversion rate was quantified via an interval-censored survival model using an exponential distribution. The table below showed the number of reversion and total number observed at baseline for each TDRM. NRTI resistance mutations showed the largest variability that included both the fastest (184V) and the slowest (215D) reverting TDRM.

In total, 21 TDRM were analyzed. One mutation (190A of NNRTI) was excluded because we observed no reversion at all from the studied patients and three mutations (101E, 181C, 210W) were further excluded because they were located in the HLA epitopes (see Methods). Thus we could obtain reversion rates for 17 TDRM (Fig. 1). Among them, 10 were mutations associated with resistance to NRTI, 6 to PI, and 1 to NNRTI. The quantified linear reversion rate showed that persistence time varied strongly among mutations. Among three drug classes, NRTI mutations showed the largest variability. Both the fastest and the slowest reversion rates, 174.0/100-person-years [confidence interval = 51.4, 588.8] from 184V and 2.7/100-person-years [0.7, 10.9] from 215D, respectively, belonged to this drug class.

### Predicted fitness cost is associated with TDRM persistence

**Fig 2:**
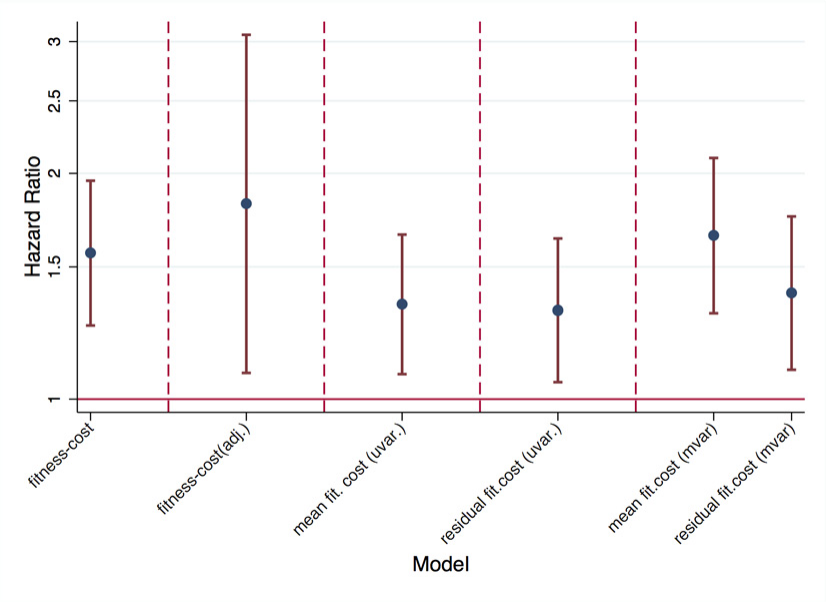
Impact of fitness cost on reversion rates. In unadjusted survival analysis (“fitness cost”), in survival analysis adjusted for type of mutation (“fitness cost adj.”). Impact of mean fitness cost and residual fitness cost in univariable analysis (“uvar.”) and in multivariable analysis including both mean and residual fitness cost (“mvar.”).

We found that reversion rates were associated significantly with the predicted fitness costs of resistance mutations (Fig. 2). Specifically, the survival analysis with predicted fitness cost as an explanatory variable yielded that reversion rates increased by a factor 1.6[1.3,2.0] (p<0.001) if fitness is increased by one standard deviation. Thus predicted fitness has a considerable and highly significant impact on reversion rates. Since this analysis included different fitness costs of mutations, each in at least five patients, the observed effect of fitness can be caused by two mechanisms: On the one hand, by overall differences in costs among mutations (“main effects”) and, on the other hand, by different costs of the same mutation in different backgrounds (“epistatic effects”). In order to distinguish between these two effects, we further analyzed the data with two alternative approaches:

In the first approach, we still used predicted fitness cost as the explanatory variable but adjusted for the identity of the resistance mutation (i.e. the type of resistance mutation was included as a categorical variable). In this approach, the estimated effect of fitness corresponded to the impact of fitness within a given type of mutation. Since this approach introduced 17 variables for 264 data points and 62 events (and hence carries the risk of over-parameterization), we considered an alternative second approach, which only included two parameters. Specifically, we divided fitness cost into two components: the mean fitness cost of a mutation (across backgrounds) and the residual fitness cost, which is given as the difference between the predicted fitness cost in a given background and the mean fitness cost. In the first approach, reversion rate was increased by a factor 1.8[1.1,3.1] (p<0.001) if fitness cost was increased by one standard deviation (after adjusting for type of mutation). In the second approach, both mean fitness cost and residual fitness cost increased the reversion rate significantly by a factor 1.7[1.3, 2.1] (p<0.001) and 1.4[1.1,1.8] (p = 0.007) per standard deviation, respectively. Thus our models predict that a typical difference in fitness cost among resistance mutations (i.e. one standard deviation of the fitness costs observed in our data set), causes a 40%-80% increase in the rate with which resistance mutations revert. Moreover, both approaches showed that both types of fitness cost (different overall costs of drug resistance mutations, and different costs in different backgrounds) are associated with higher reversion rates.

These multivariable models also showed that, as can be expected, reversion occurs much faster if a given TDRM is present as a mixture (see Methods, Table 2).

**Table 2:** Hazard ratios (HR) reported in univariable and multivariable models.

	Univar. HR (95% CI)	p	Multivar. HR (95% CI)	p
Mean fitness cost	1.31 (1.05,1.64)	0.015	1.65 (1.30,2.10)	<0.001
Residual fitness cost	1.34 (1.08,1.66)	0.008	1.38 (1.09,1.75)	0.007
TDRM present as mixture	9.71 (5.87,16.1)	<0.001	12.3 (7.22, 20.1)	<0.001

## Discussion

In this study we investigated the differential persistence behaviors of TDRM in the absence of drug pressure and analyzed the association of the reversion rate with the predicted fitness cost of a given mutation. We used an interval-censored survival model to quantify the reversion rate of each mutation that was at least harbored by five individuals at baseline. We observed that the reversion rate of individual mutations varied substantially. Moreover, the reversion rates were significantly associated with the differential fitness costs of the TDRM: We showed that both the fitness-cost differences among mutations and among viral genetic backgrounds for the same mutation contributed to the variation in reversion rates. Thus, the novelty of this study is that we compared in total 17 TDRM from patients in a single cohort and could associate the persistence times with fitness costs of mutations predicted by a machine-learning model. An additional strength of this study is the high frequency and the number of resistance tests performed per patient.

Our results were consistent with most studies showing that M184V disappeared rapidly [15,21,29] whereas most thymidine analogue associated mutations (TAMs: 41L, 67N, 70R, 215Y, 219Q) disappeared at a slower rate [21,29,30] with the exception of 70R and 215Y. It is known however that 215Y has a high impact on fitness [21] and is rapidly replaced by intermediate 215S or atypical variants 215C/D [31]. Additionally, the fitness cost of 70R was shown to be higher when combined with other mutations *in vitro* [21,24]. This could explain the observed high reversion rate of 70R regardless of its low fitness cost because in our data set 7 from 11 patients harboring 70R had at least one other mutation. Our data showed that most TDRM to PI reverted more rapidly, compared to NRTI mutations.

From a more general perspective our findings have important implications for understanding the epidemic spread of drug-resistant pathogens. One of the general problems with drug resistance is that it can be quickly selected by drug pressure, but upon transmission it reverts only slowly if at all in the absence of drug pressure [32]. The intuition behind this is that drugs cause an enormous reduction in the replicative capacity of wild-type virus and hence lead to a strong relative fitness benefit for resistant mutants. By contrast, the fitness cost in the absence of drugs is typically weak. Our results highlight the large variability in reversion rates and the central role of fitness cost in governing the speed of reversion in the *in vivo* setting within the SHCS. In particular, they show that the genetic background of a resistance mutation substantially modulates the fitness cost and thereby the reversion rate of the mutation. This implies heritable variation in the fitness cost of resistance and thereby the danger that such fitness costs are reduced by evolutionary selection, i.e. mutations in genetic backgrounds causing lower fitness cost will have larger chances to spread to other patients and hence may dominate the population in the long run. Assessing the impact of the genetic background on reversion rates is central for understanding the spread of antimicrobial resistance in general. For example, theoretical models and *in vitro* evidence suggest a crucial role of compensatory mutations in boosting antibiotic resistance for a broad range of bacterial pathogens [33]. However, real-world epidemiological evidence for an impact of the genetic backgrounds found in natural pathogen populations on reversion of resistance in patients is largely lacking. In this context our approach offers a proof of principle for using machine learning approaches to bridge the gap between epidemiological data on resistance reversion and *in vitro* fitness measurements and thereby to address this crucial issue.

In the context of HIV epidemiology in Switzerland, such a scenario of mutation evolution can be probably prevented by the good surveillance and the early treatment of HIV-infected individuals, implying that resistant strains have only limited opportunity to cause new infections and hence to select backgrounds with lower fitness cost. By contrast, this scenario is a very real danger in settings with poorer surveillance and hence ampler opportunities for resistant viruses to spread. In those settings evolution might indeed successfully act on the variation of fitness costs and lead in the long term to resistant viruses with a low fitness cost.

Previous work [18] has assessed fitness costs of some antiretroviral resistance mutations *in vitro* by site directed mutations (SDM). Since these studies did not consider the impact of different genetic backgrounds, we can only compare the average fitness cost of a mutation determined by our method with the fitness costs determined by SDM. This comparison reveals a good qualitative but not perfect agreement to our estimates with SDM data (as summarized in [18]). Estimates were available in both data sets for the RT mutations 184V, 70R, 41L, 103N, and 215Y; in agreement with [18] we found a high fitness cost for 184V (1.8 standard deviations above mean fitness cost = +1.8s.d) and a moderate fitness cost for 70R, 41L, and 103N (+0.58 s.d., −0.16 s.d., and +0.48 s.d., respectively). In agreement with [18] we also found moderate fitness costs for 210W and 181C (−0.85 s.d. and −0.69 s.d. respectively), which were excluded from our analysis because they lie in HLA epitopes and disrupt binding. The main discrepancy was found for 215Y, where our methods predicted low fitness costs (−0.86 s.d.) in contrast to the SDM data [18]. The fact that reversion rates are high for this mutation indicates that our estimator has underestimated the real fitness cost of this mutation. This failure may be also related to the complexity of the mutational pathways at this position, which may have been oversimplified by our approach (in which we do not distinguish which amino acid a TDRM reverts to). This deviation is also not surprising since the computational predictor underlying our approach is not perfect (42% of deviance in *in vitro* fitness were explained in [27]). Overall this comparison thus validates our method but also reveals that there is potential for improvement and hence our approach should be best viewed as a proof of principle of using machine-learning approaches in conjunction with *in vitro* fitness measurements to assess reversion of TDRM *in vivo*.

This assessment of the fitness predictor is confirmed by considering the quality of fit of the different models summarized in Fig. 2: Starting from an interval-censored survival model without explanatory variables, adding the information of whether a given TDRM is present as a mixture reduces the model deviance by 22%. Adding TDRM-fitness as an explanatory variable reduces the model’s deviance by a further 9%. If we separate fitness cost into the mean fitness cost of a given mutation type and the corresponding residual fitness cost (as in Fig. 2), this 9% results from a 6% of deviance-reduction explained by the mean fitness cost and 3% by the residual fitness cost. This indicates an important role of fitness for TDRM reversion; especially given that, firstly, the fitness predictor used here is not perfect (it explains 42% of deviance of *in vitro* replicative capacity [27]) and that, secondly, being a mixture implies that a nucleotide has already started to revert and hence the corresponding variable represents a very strong determinant of reversion. Finally, these numbers suggest that the differential fitness-costs of the same mutation in different genetic backgrounds contribute half as much to the population-level variability in reversion than different fitness-costs of different mutations. Given the well-described and strong differences in reversion rates across mutation types this therefore implies an important role of the genetic background. However, these fractions of deviance explained by our predicted fitness costs imply that reversion rates also depend on other factors not captured by *in vitro* replicative capacity. This includes interactions between host-viral factors such as HLA escape. Even though we excluded TDRMs known to mediate CTL escape (see Methods), it is likely that this does not encompass all such escape mutations or more generally all mutations that affect the interaction of a virus with a given patient’s immune system.

Our study had several limitations. One of the limitations of this study was the lack of information before the first GRT was performed. More specifically, we could not determine how long a TDRM had already persisted before the first GRT. We studied the reversion of TDRM from the baseline GRT instead of the infection date of a patient because an exact infection date was not known for most of the patients and because GRTs at infection time are typically not available. This approach increased the sample size considerably in exchange for missing some TDRM that had reverted before the first GRT was performed. This could explain why K65R or T215F, which are known to revert rapidly, were not identified in our study. The fast reverting TDRM such as M184V were either missed or detected right after the infection by GRT, thus the estimated reversion rates were not altered to a large extent and only the sample size may be lower. Another limitation was that around 40% (67 / 168) of patients carried > 1 TDRM at baseline. Although combinations of mutations could modulate the fitness costs substantially [21], causing that a given mutation has varying fitness costs when having different genetic backgrounds, the number of mutations detected at the first GRT was not found to be associated with the reversion of TDRM [29]. Additionally we adjusted for different genetic backgrounds including the residual fitness costs in our model and still found positive associations of reversion rates with average fitness costs.

In conclusion, our study demonstrated that TDRM showed substantial variation in reversion rates, which were positively associated with the fitness costs these mutations had in their genetic background.

## Supporting Information

S1 Table Observed frequency at baseline and number of reversion from mutations ever observed. (DOCX)
