# Supplementary material for: Persistence of Transmitted HIV-1 Drug Resistance Mutations Associated with Fitness Costs and Viral Genetic Backgrounds
Source: PLoS Pathog. 2015 Mar 23;11(3):e1004722. doi: 10.1371/journal.ppat.1004722 (PMC4370492; doi:10.1371/journal.ppat.1004722)
Supplement: S1 Table — (DOCX) [file ppat.1004722.s001.docx]

S1 Table. Observed frequency at baseline and number of reversion from mutations ever observed

| Drug Class | Mutation | Number of reversion / Observed frequency at baseline (percentage) |
| --- | --- | --- |
| NRTI | 41L | 9 / 45 (20.0) |
|  | 65R | 2 / 3 (66.7) |
|  | 67G | 1 / 5 (20.0) |
|  | 67N | 3 / 14 (21.4) |
|  | 69D | 1 / 1 (100.0) |
|  | 70E | 1 / 1 (100.0) |
|  | 70R | 7 / 11 (63.6) |
|  | 74I | 1 / 1 (100.0) |
|  | 74V | 1 / 3 (33.3) |
|  | 75A | 1 / 1 (100.0) |
|  | 77L | 0 / 1 (0.0) |
|  | 116Y | 1 / 2 (50.0) |
|  | 151M | 0 / 1 (0.0) |
|  | 184I | 1 / 1 (100.0) |
|  | 184V | 5 / 9 (55.6) |
|  | 210W | 4 / 14 (28.6) |
|  | 215C | 6 / 8 (75.0) |
|  | 215D | 4 / 24 (16.7) |
|  | 215E | 1 / 3 (33.3) |
|  | 215F | 1 / 4 (25.0) |
|  | 215I | 2 / 1 (200.0) |
|  | 215S | 8 / 25 (32.0) |
|  | 215V | 0 / 1 (0.0) |
|  | 215Y | 8 / 10 (80.0) |
|  | 219E | 2 / 3 (66.7) |
|  | 219Q | 2 / 15 (13.3) |
|  | 219R | 3 / 4 (75.0) |

| Drug Class | Mutation | Number of reversion / Observed frequency at baseline (percentage) |
| --- | --- | --- |
| PI | 23I | 1 / 1 (100.0) |
|  | 24I | 0 / 2 (0.0) |
|  | 30N | 1 / 2 (50.0) |
|  | 46I | 5 / 16 (31.2) |
|  | 46L | 5 / 8 (62.5) |
|  | 47V | 3 / 3 (100.0) |
|  | 48V | 0 / 1 (0.0) |
|  | 50V | 1 / 1 (100.0) |
|  | 53L | 0 / 1 (0.0) |
|  | 54M | 0 / 1 (0.0) |
|  | 54V | 1 / 6 (16.7) |
|  | 73C | 1 / 2 (50.0) |
|  | 73S | 2 / 2 (100.0) |
|  | 76V | 1 / 1 (100.0) |
|  | 82A | 2 / 6 (33.3) |
|  | 82L | 0 / 1 (0.0) |
|  | 82T | 1 / 1 (100.0) |
|  | 83D | 0 / 1 (0.0) |
|  | 84V | 1 / 3 (33.3) |
|  | 85V | 1 / 5 (20.0) |
|  | 88D | 1 / 2 (50.0) |
|  | 90M | 2 / 21 (9.5) |

| Drug Class | Mutation | Number of reversion / Observed frequency at baseline (percentage) |
| --- | --- | --- |
| NNRTI | 101E | 2 / 5 (40.0) |
|  | 101P | 0 / 1 (0.0) |
|  | 103N | 9 / 35 (25.7) |
|  | 103S | 0 / 2 (0.0) |
|  | 179F | 1 / 1 (100.0) |
|  | 181C | 2 / 8 (25.0) |
|  | 188L | 1 / 3 (33.3) |
|  | 190A | 0 / 7 (0.0) |
|  | 225H | 0 / 1 (0.0) |
